# Muscle-lung crosstalk in COPD: how exercise training modulates protein metabolism and inflammation in experimental COPD models

**DOI:** 10.3389/fimmu.2026.1870091

**Published:** 2026-09-25

**Authors:** Asghar Abbasi, Rodolfo P. Vieira, Fernanda Degobbi T. Q. S. Lopes

**Affiliations:** 1 Respiratory Research Center, The Lundquist Institute for Biomedical Innovation at Harbor-UCLA Medical Center, Torrance, CA, United States; 2 Division of Respiratory Critical Care Physiology and Medicine, Harbor-UCLA Medical Center, Torrance, CA, United States; 3 College of Health Sciences, The University of Texas at El Paso, El Paso, TX, United States; 4 Laboratory of Pulmonary and Exercise Immunology (LABPEI), Evangelical University of Goias (UniEvangelica), Anápolis, Brazil; 5 Department of Research and Development, Brazilian Institute of Teaching and Research in Pulmonary and Exercise Immunology (IBEPIPE), Anápolis, Brazil; 6 Laboratory of Experimental Therapeutics (LIM20), Department of Clinical Medicine, School of Medicine, University of Sao Paulo, Sao Paulo, Brazil; 7 Thoracic Surgery Research Laboratory (LIM61), Division of Thoracic Surgery, Instituto do Coracao do Hospital das Clinicas da Faculdade de Medicina da Universidade de Sao Paulo, Sao Paulo, Brazil

**Keywords:** biomarkers, COPD model, exercise training, inflammatiom, lung, muscle, protein metabolism

## Abstract

Chronic obstructive pulmonary disease (COPD) is a multisystem inflammatory disease in which skeletal muscle dysfunction contributes substantially to exercise intolerance, reduced functional capacity, and poor clinical outcomes. Increasing evidence suggests that bidirectional communication between skeletal muscle and the lung plays an important role in disease progression and in mediating the beneficial effects of exercise training. This review summarizes current knowledge of muscle-lung crosstalk in cigarette smoke (CS)-induced experimental models of COPD, while incorporating complementary evidence from other experimental models where relevant to support mechanistic interpretation. We discuss the molecular pathways linking pulmonary inflammation to skeletal muscle dysfunction, including systemic inflammation, oxidative stress, mitochondrial dysfunction, impaired protein turnover, and metabolic dysregulation. We further examine how skeletal muscle may influence pulmonary biology through the release of myokines, metabolites, extracellular vesicles, and mitochondrial-derived signaling molecules, highlighting their potential roles in inter-organ communication. Particular emphasis is placed on the capacity of exercise training to modulate these pathways by improving muscle proteostasis, mitochondrial function, systemic inflammatory status, and pulmonary outcomes in experimental model of COPD. We also discuss important considerations for interpreting findings across experimental models, including differences in cigarette smoke exposure paradigms, exercise timing, and distinctions between locomotor and respiratory muscles. Finally, we identify key gaps in current knowledge and outline future experimental strategies needed to define the molecular mechanisms underlying muscle-lung crosstalk and to facilitate translation of these findings into exercise-based therapeutic approaches for COPD.

## 1 Introduction

### 1.1 COPD as a multisystem disease

Chronic obstructive pulmonary disease (COPD) is a leading cause of morbidity and mortality worldwide and is characterized by persistent respiratory symptoms and airflow limitation resulting from airway and/or alveolar abnormalities. Its clinical consequences extend beyond the lung. Among its major extrapulmonary manifestations, skeletal muscle dysfunction, particularly affecting the locomotor muscles, is characterized by reduced muscle mass and strength, impaired oxidative capacity, fiber-type remodeling, and increased fatigability. These abnormalities are independently associated with exercise intolerance, impaired quality of life, hospitalization, and mortality (1–3). The concept of COPD as a systemic disease is further supported by the frequent coexistence of systemic inflammation and multiple extrapulmonary manifestations (4).

The mechanisms underlying skeletal muscle dysfunction in COPD are multifactorial and incompletely understood. Physical inactivity and deconditioning are important contributors, but chronic or episodic systemic inflammation, hypoxemia, nutritional imbalance, oxidative stress, cigarette smoke-related toxicity, corticosteroid exposure, and dysregulated anabolic and catabolic signaling all contribute to disease progression (2, 3). Collectively, these interconnected processes support an inter-organ framework in which pulmonary injury influences skeletal muscle through systemic inflammatory, metabolic, and neurohumoral signaling, while skeletal muscle communicates with the lung through the release of bioactive mediators capable of modulating pulmonary immune, inflammatory, and metabolic responses.

### 1.2 Experimental animal models of COPD: utility and limitations

No single animal model fully recapitulates the clinical and pathological complexity of human COPD. Consequently, model selection should be guided by the specific biological question being addressed, and mechanistic conclusions should be interpreted within the context of the phenotype reproduced by each model (**Figure 1**). Chronic cigarette smoke (CS) exposure serves as the primary experimental model discussed in this review because it most closely reflects the principal etiological factor underlying human COPD. Depending on the species, strain, exposure protocol, and duration, chronic CS exposure reproduces key features of the disease, including airway inflammation, emphysematous remodeling, oxidative stress, exercise intolerance, and skeletal muscle dysfunction (5–8).

**Figure 1:**
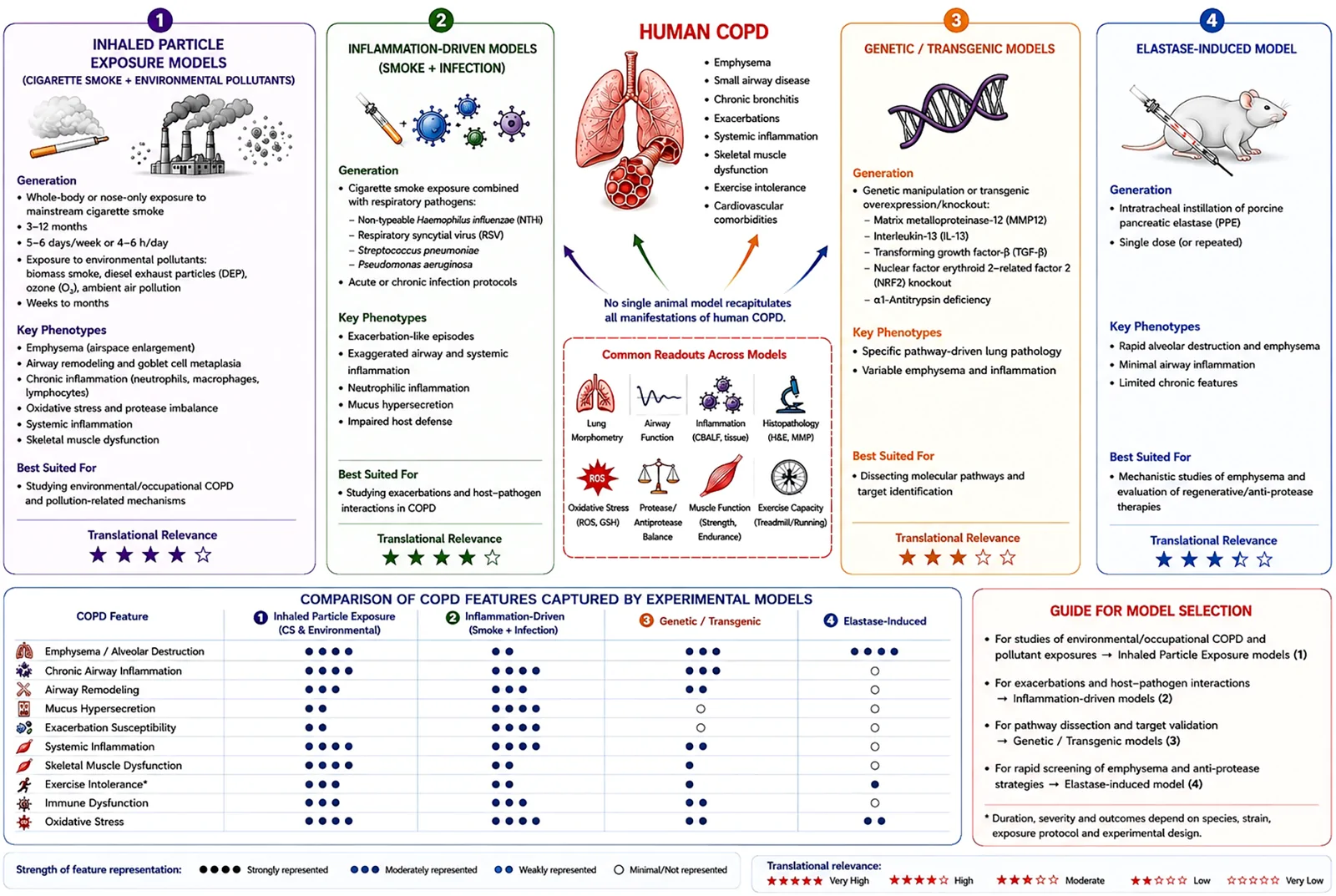
Experimental animal models of COPD: generation, disease phenotypes, and utility for research.

Considerable heterogeneity exists among CS exposure protocols. Short-term or sub-chronic exposure (typically several weeks to approximately 3 months) predominantly induces pulmonary inflammation, oxidative stress, and early molecular alterations before stable emphysematous remodeling develops. In contrast, prolonged exposure (commonly 3–12 months in rodents) more consistently reproduces persistent structural lung damage and systemic manifestations of COPD. Furthermore, differences in exposure methodology, including whole-body versus nose-only exposure, mainstream versus sidestream smoke, particulate concentration, animal sex, age, genetic background, and the interval between the final smoke exposure and outcome assessment, can substantially influence disease severity and experimental outcomes. These methodological differences should therefore be carefully considered when comparing studies and evaluating their translational relevance (5, 9–12).

Several experimental variables also influence extrapulmonary manifestations of COPD and may confound the interpretation of skeletal muscle outcomes. Chronic CS exposure can reduce spontaneous physical activity, alter food intake and body weight, increase physiological stress, and induce varying degrees of hypoxemia. In addition, housing conditions, ambient temperature, nutritional status, and animal handling can independently affect skeletal muscle mass, metabolism, and function. Consequently, skeletal muscle abnormalities observed in smoke-exposed animals should not be attributed solely to lung-derived inflammatory signaling without accounting for these potential confounding factors. Studies incorporating assessments of physical activity, nutritional intake, body composition, arterial oxygenation, and local skeletal muscle inflammatory and redox pathways provide a more robust mechanistic framework for interpreting muscle dysfunction.

Although chronic CS exposure remains the most clinically relevant model for studying smoking-induced COPD, complementary experimental models provide important mechanistic insights. Elastase-induced emphysema rapidly and reproducibly produces alveolar destruction but does not recapitulate the chronic biological effects of cigarette smoke exposure and generally elicits a less sustained systemic inflammatory response (13–16). Lipopolysaccharide (LPS)-based and repeated inflammatory challenge models are particularly valuable for investigating inflammation-driven pulmonary and extrapulmonary responses, whereas combined CS and bacterial or viral infection models more closely reflect COPD exacerbations and impaired host defense mechanisms (17–20). Environmental pollutant exposure and genetically modified animal models further facilitate investigation of specific molecular pathways contributing to disease pathogenesis (21–29). Rather than representing alternative models, these approaches provide complementary experimental systems that address biological questions not readily resolved using chronic CS exposure alone.

Exercise interventions introduce an additional source of experimental heterogeneity. Aerobic treadmill exercise, voluntary wheel running, interval training, and other exercise paradigms differ in exercise intensity, physiological stress, and patterns of skeletal muscle recruitment, all of which may influence pulmonary and systemic adaptations (30–35). Equally important is the timing of exercise relative to disease development. In many experimental studies, exercise is initiated before or during CS exposure, thereby evaluating its preventive effects on disease development. In contrast, therapeutic paradigms introduce exercise after COPD-like pathology has been established and therefore more closely resemble pulmonary rehabilitation in clinical practice. For example, the Toledo-Arruda model implemented treadmill exercise throughout the period of CS exposure and should be interpreted primarily as a preventive intervention (36), whereas Krüger et al. initiated exercise during the final two months of an eight-month CS exposure protocol, providing stronger evidence that exercise can attenuate established systemic and skeletal muscle abnormalities (37). Throughout this review, preventive and therapeutic exercise paradigms are clearly distinguished to facilitate interpretation of the available evidence.

### 1.3 Scope and evidence framework for this review

This review focuses primarily on experimental models of COPD induced by chronic cigarette smoke (CS) exposure, as these models most closely reproduce the principal etiological factor underlying human COPD and provide the strongest experimental platform for investigating muscle-lung crosstalk. However, because the molecular mechanisms governing inter-organ communication remain incompletely understood, evidence from additional experimental systems is incorporated where it provides complementary mechanistic insight. These include alternative models of COPD and emphysema, mechanistic *in vitro* studies, and human studies examining skeletal muscle, lung tissue, or responses to exercise interventions.

Throughout this review, mechanistic conclusions are based preferentially on studies demonstrating direct biological interactions between skeletal muscle and the lung. Findings derived from healthy exercise models or non-COPD inflammatory conditions are included only when they provide relevant biological context or support potential mechanisms that have yet to be investigated in COPD. Importantly, associations between circulating mediators and physiological outcomes are not considered evidence of inter-organ communication in the absence of experiments demonstrating causality, such as tissue-specific genetic manipulation, pharmacological inhibition, receptor blockade, source tracing, or transfer studies.

### 1.4 Bidirectional muscle-lung crosstalk

Bidirectional communication between the lung and skeletal muscle represents a fundamental component of the systemic manifestations of COPD. In the lung-to-muscle direction, chronic pulmonary injury promotes a systemic milieu characterized by persistent inflammation, oxidative stress, hypoxemia, neurohumoral dysregulation, and reduced physical activity. Collectively, these alterations disrupt skeletal muscle homeostasis by suppressing anabolic signaling, activating proteolytic pathways, impairing mitochondrial function, and ultimately reducing muscle mass, strength, and endurance.

Conversely, skeletal muscle functions as an endocrine organ that releases a diverse array of bioactive mediators, including myokines, cytokines, metabolites, and extracellular vesicles (EVs), into the circulation during contraction and in response to exercise training. Many of these mediators regulate immune, metabolic, and inflammatory processes in distant organs, supporting the concept that exercise-induced adaptations in skeletal muscle may influence pulmonary physiology and immune responses (38–41) (**Figure 2**).

**Figure 2:**
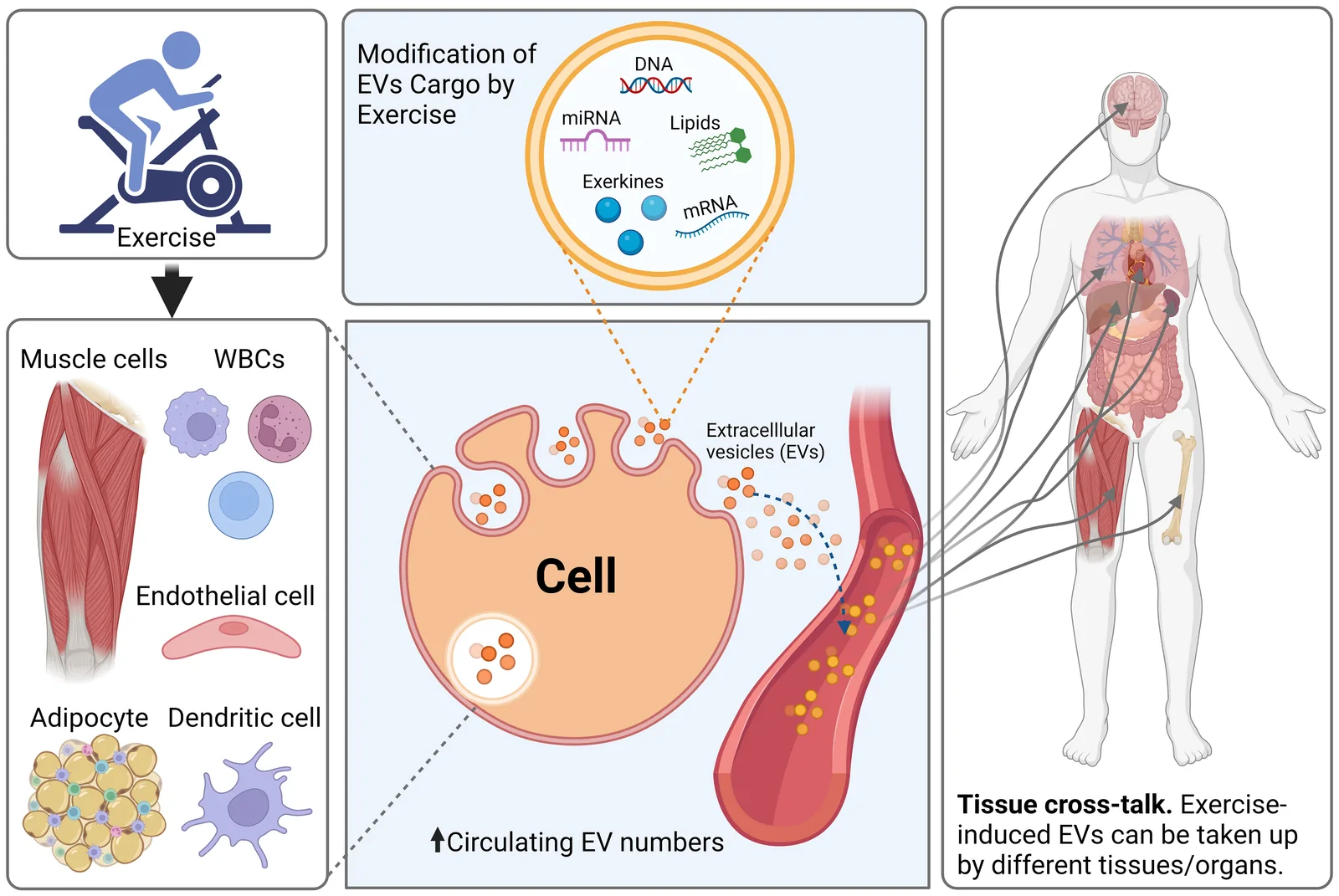
Exercise-associated extracellular vesicles as candidate mediators of inter-organ communication. Exercise changes EV release and cargo, but circulating EVs arise from multiple cell types; tissue-of-origin and causal uptake by the lung require direct experimental validation.

Importantly, the presence of circulating mediators does not necessarily identify their tissue of origin. Molecules such as IL-6, IL-10, IL-1 receptor antagonist (IL-1ra), TNF-α, extracellular vesicles, and mitochondrial-derived products are produced by multiple cell types, including immune cells, adipose tissue, vascular endothelium, airway epithelium, and skeletal muscle. Throughout this review, careful distinction is therefore made between the biological compartment in which a mediator is measured (e.g., skeletal muscle, plasma or serum, bronchoalveolar lavage fluid, or lung tissue) and the cellular source responsible for its production.

### 1.5 Exercise training as a modifier of the axis

Exercise training is a cornerstone of pulmonary rehabilitation and represents one of the most effective non-pharmacological interventions for patients with COPD, consistently improving exercise capacity, dyspnea, and health-related quality of life (42–44). Although the clinical benefits of exercise are well established, the molecular mechanisms underlying these adaptations remain incompletely understood. In skeletal muscle, exercise promotes mitochondrial biogenesis, enhances oxidative capacity and capillarization, improves neuromuscular function, and restores the balance between anabolic and catabolic signaling, thereby improving muscle performance and exercise tolerance. In experimental models of COPD, exercise has also been shown to attenuate pulmonary inflammation, oxidative stress, and structural lung injury under specific experimental conditions (30, 34, 36, 37, 45–47) (**Table 1**).

**Table 1:** Representative experimental studies evaluating exercise training in COPD models, including exercise modality, timing, and major muscle/systemic and pulmonary outcomes.

Study	COPD model/exposure	Exercise modality/protocol	Timing	Major exercise-related effects	Interpretation
Toledo et al. (30)	C57BL/6 mouse; CS CS 30 min/day, 5 days/week, 24 weeks	Moderate treadmill; 50% maximal speed, 60 min/day, 5 days/week, 24 weeks	Concurrent/preventive	Muscle/systemic: Improved exercise capacity Pulmonary: Reduced BAL ROS and pulmonary injury; preserved antioxidant/anti-inflammatory responses	Long-duration concurrent training; prevention rather than treatment of established disease
Toledo-Arruda et al. (31)	C57BL/6 mouse; CS CS with 4-, 8-, or 12-week time points	Moderate treadmill; 50% maximal speed, 2 x 30 min/day, 5 days/week	Concurrent/preventive	Muscle/systemic: Improved functional capacity; attenuated peripheral-muscle oxidative/cachexia-related changes Pulmonary: Pulmonary effects characterized in companion/time-course work	Defines time-dependent muscle adaptation during concurrent CS and training
Henriques et al. (32)	BALB/c mouse; elastase emphysema PPE 0.1 IU intratracheally weekly for 4 weeks	Moderate treadmill; 8–12 m/min, 5% grade, 30 min/day, 3 days/week, 4 weeks (~65-70% VO2max)	Therapeutic; after emphysema established	Muscle/systemic: Cardiorespiratory performance assessed Pulmonary: Reduced hyperinflation, remodeling and TNF-alpha; improved lung mechanics/VEGF; reduced RV enlargement	Therapeutic exercise in an elastase model
Rodrigues Brandao-Rangel et al. (34)	C57BL/6 mouse; CS CS for 90 days	Treadmill; 50% maximal velocity, 25° incline, 60 min/day, 5 days/week, 4 weeks	Therapeutic/late concurrent; days 61-90	Muscle/systemic: Reduced circulating inflammatory-cell/cytokine responses Pulmonary: Reduced BAL inflammation and inflammatory mediators; increased IL-10; modulated pulmonary STAT3	Exercise introduced after 60 days of CS; stronger disease-modification design
Krüger et al. (37)	C57BL/6J mouse; mainstream CS CS 6 h/day, 5 days/week, 8 months	Treadmill exercise, 5 days/week during final 2 months	Therapeutic/late concurrent	Muscle/systemic: Restored VO2max/running speed; reduced systemic inflammation and muscle wasting; improved IGF-1/mTOR and oxidative phenotype Pulmonary: Pulmonary structural outcomes not primary endpoints	Strong evidence for reversal of established systemic and skeletal-muscle abnormalities
Vieira Ramos et al. (71)	Mouse; chronic CS CS 30 min/day, 5 days/week, 24 weeks	Moderate treadmill; 50% maximal exercise capacity, 60 min/day, 5 days/week, 24 weeks	Concurrent/preventive	Muscle/systemic: Prevented diaphragm wasting; increased Nrf2/Hmox1; reduced MMP-2/MMP-9 activity Pulmonary: Lung outcomes not primary endpoints	Respiratory-muscle-specific evidence; should not be generalized to locomotor muscle
Yu et al. (45)	Mouse; environmental CS Exercise for 8 weeks; CS introduced during final 4 weeks	Aerobic exercise training for 8 weeks	Preconditioning + concurrent/preventive	Muscle/systemic: Not a primary muscle study Pulmonary: Reduced bronchoalveolar-capillary permeability, inflammatory-cell infiltration, epithelial thickening, inflammatory mediators and NF-kappaB activation	Demonstrates protection from subsequent environmental CS exposure
Toledo-Arruda et al. (36)	C57BL/6J mouse; CS CS 2 x 30 min/day, 5 days/week for 4, 8, or 12 weeks	Moderate treadmill; 50% maximal speed, 25% incline, 2 x 30 min/day, 5 days/week	Concurrent/preventive	Muscle/systemic: Improved aerobic conditioning; muscle cytokine/redox adaptations Pulmonary: Reduced BAL inflammation; improved antioxidant defenses and lung mechanics; attenuated emphysema at 12 weeks	Time-course evidence showing protection develops with training adaptation
Kubo et al. (47)	Mouse; CS Daily CS for 12 weeks	Treadmill; 18 m/min, 0° incline, 30 min/day, 5 days/week, 12 weeks; before daily CS	Concurrent/preventive	Muscle/systemic: Increased circulating irisin Pulmonary: Reduced emphysema/inflammation with increased pulmonary Nrf2/HO-1 signaling	Supports exercise-irisin-Nrf2 association; does not establish muscle-derived irisin causality
Suehiro et al. (166)	WT and VAChT-deficient mice; CS CS 30 min, 2x/day, 5 days/week, 12 weeks	Aerobic treadmill; 60 min/day, 5 days/week, 12 weeks	Concurrent/preventive	Muscle/systemic: Not a primary muscle study Pulmonary: Reduced emphysema and mononuclear-cell infiltration in WT mice; cholinergic dependence for selected effects	Provides mechanistic evidence for cholinergic contribution to pulmonary exercise effects
Hassel et al. (167)	A/JOlaHsd mouse; CS Mainstream CS 6 h/day, 5 days/week, 14 weeks	HIIT treadmill; 25° incline, 4-min bouts at 85-90% peak work alternating with 2 min at 50-60%; 60 min/day, 5 days/week, 4 weeks	Post-exposure/therapeutic	Muscle/systemic: Improved VO2peak Pulmonary: Reversed RV dysfunction; reduced pulmonary-vessel muscularization; no reversal of alveolar enlargement	Shows cardiovascular/pulmonary vascular adaptation despite persistent structural emphysema
Bowen et al. (168)	Mouse; whole-body CS CS for 20 weeks	High-intensity interval treadmill training for 6 weeks	During prolonged CS exposure	Muscle/systemic: Improved exercise capacity; attenuated diaphragm dysfunction, oxidative stress and protein degradation Pulmonary: No overt airway destruction in this model	Demonstrates extrapulmonary benefit independent of measurable reversal of lung destruction
Moreno et al. (169)	Wistar rat; secondary CS 4 cigarettes, 30 min, 2x/day, 5 days/week, 16 weeks	Progressive-load resistance training; 4 ladder climbs/day, 5 days/week, 16 weeks	Concurrent/preventive	Muscle/systemic: Preserved gastrocnemius mitochondrial respiration and OXPHOS proteins Pulmonary: Pulmonary outcomes not assessed	Supports resistance-training effects on skeletal-muscle mitochondrial homeostasis; cannot establish muscle-lung signaling

Collectively, these observations support the concept that exercise exerts coordinated effects on both skeletal muscle and the lung. However, the molecular mediators responsible for this inter-organ communication remain incompletely defined. While exercise-induced adaptations in skeletal muscle are well established, the extent to which individual muscle-derived factors directly contribute to pulmonary protection has yet to be fully elucidated. Identifying the signaling pathways that mediate these systemic adaptations will be essential for understanding the biological basis of muscle-lung crosstalk and for developing targeted therapeutic strategies in COPD.

## 2 Lung-to-muscle signaling in COPD

### 2.1 Pulmonary injury and systemic signaling

Chronic cigarette smoke (CS) exposure initiates a complex inflammatory response characterized by activation of airway epithelial cells, alveolar macrophages, neutrophils, and other innate immune populations. These responses promote the release of proteases, generation of reactive oxygen species (ROS), activation of NF-κB-dependent inflammatory signaling, extracellular matrix degradation, and progressive emphysematous remodeling (48–56). Mitochondrial dysfunction within pulmonary structural and immune cells further amplifies oxidative stress while impairing macrophage phagocytosis and efferocytosis, thereby sustaining chronic pulmonary inflammation (57–63) (**Figure 3**).

**Figure 3:**
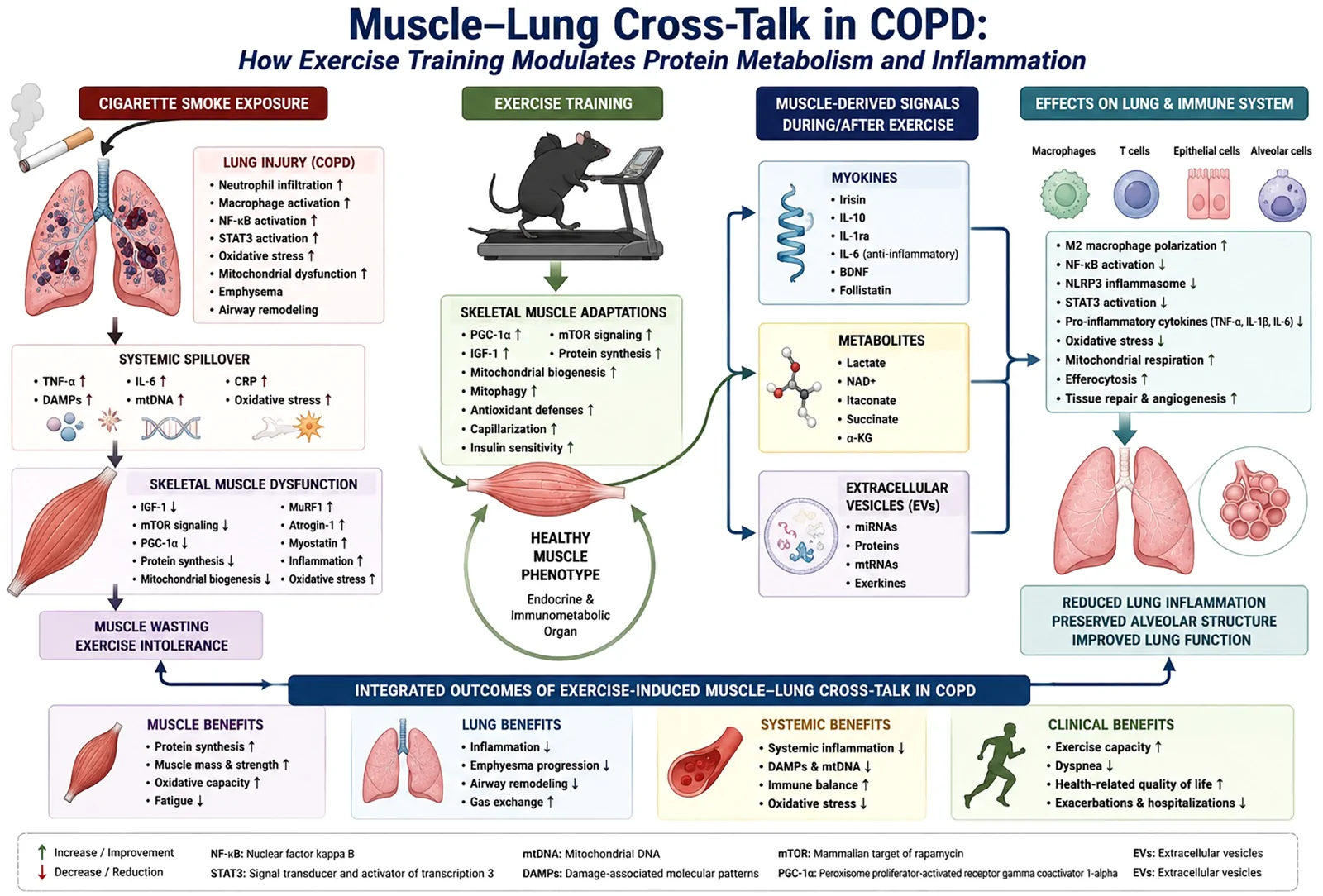
Evidence-weighted framework for bidirectional muscle-lung crosstalk in COPD. Cigarette-smoke-related pulmonary damage can influence locomotor muscle through systemic inflammatory, redox, hypoxemic, and behavioral pathways. Exercise improves skeletal-muscle phenotype and changes circulating myokines, metabolites, and EVs that may feedback to the lung. Solid mechanistic arrows should be interpreted as established biological routes at the pathway level; mediator-specific causal links in COPD remain variably supported and are summarized in **Table 2**.

**Table 2:** Candidate mediators of muscle-lung crosstalk and current evidence strength.

Mediator	Direction/source	Exercise relationship	Evidence base	Current strength	Key falsifiable experiment
IL-6	Muscle/immune/multiple tissues -> systemic	Exercise-responsive; COPD inflammation	Human exercise and broad mechanistic literature; limited source-specific COPD evidence	Limited for direct muscle->lung causality	Context- and source-dependent; avoid simple pro/anti-inflammatory label
Irisin/FNDC5	Muscle candidate -> lung	Exercise-associated increase; Nrf2	CS mouse study links exercise, irisin, Nrf2, emphysema (47); human COPD association (170)	Moderate association; limited necessity/sufficiency	Muscle-specific FNDC5 manipulation/receptor blockade
IL-10/IL-1ra	Immune/adipose/systemic -> lung	Can increase with exercise	Human/animal immune modulation; not muscle-specific (44, 110–116)	Limited as muscle-derived mediators	Identify cellular source and target tissue
Lactate	Muscle/systemic -> immune cells	Acute exercise increase	Mechanistic immune-cell data (120–124); little direct CS-COPD evidence	Indirect	Tracer and HCAR1 blockade studies in CS + exercise
NAD+/TCA metabolites	Multiple tissues -> immunometabolic targets	Exercise/metabolic remodeling	Cellular and non-COPD mechanistic evidence (125–131)	Indirect	Measure flux and target-cell pathway engagement in COPD models
Lung-derived EVs	Airway/lung -> immune/systemic targets	Altered by smoke	CSE airway epithelial EV effects on macrophages (134–136)	Moderate for local lung EV signaling; low for lung->muscle	*In vivo* source tracing and muscle uptake/function
Exercise-associated EVs	Multiple cells, including muscle -> distant organs	EV abundance/cargo change with exercise	Exercise EV literature (137–149); direct COPD lung causality sparse	Limited	Tissue-specific EV tracing, depletion, transfer
mtDAMPs	Damaged mitochondria from multiple tissues -> innate immune cells	Potentially reduced with improved mitochondrial quality	Broad DAMP biology (154–161); source-specific COPD exercise evidence sparse	Indirect	Muscle-specific tracing and receptor blockade

Experimental studies consistently demonstrate that chronic cigarette smoke exposure induces systemic alterations extending beyond the lung and promotes skeletal muscle remodeling. Depending on the duration of smoke exposure and the experimental model, mice develop reduced body weight, loss of hindlimb muscle mass, impaired muscle strength and exercise capacity, together with dysregulation of pathways controlling skeletal muscle protein homeostasis. These changes include suppression of IGF-1 signaling, activation of ubiquitin-proteasome components such as MuRF1 and atrogin-1, increased expression of inflammatory mediators including IL-6, and reduced Akt signaling, all of which favor skeletal muscle atrophy (64–66). These findings support the concept that pulmonary injury is accompanied by systemic molecular changes capable of influencing skeletal muscle remodeling.

Several mechanisms may contribute to lung-to-muscle communication during chronic CS exposure (**Figure 3**). Pulmonary inflammation is accompanied by systemic inflammatory responses that may affect skeletal muscle signaling and protein homeostasis. In experimental CS models, increased circulating inflammatory mediators have been associated with skeletal muscle inflammation, activation of NF-κB-dependent signaling, increased expression of the muscle-specific ubiquitin ligases MuRF1 and Atrogin-1, and suppression of anabolic pathways, including IGF-1 signaling (66, 67). Chronic CS exposure also induces oxidative modifications of contractile and metabolic proteins in both locomotor and respiratory muscles, providing an additional mechanism through which smoke-induced pulmonary disease may contribute to skeletal muscle dysfunction (7, 68).

Chronic pulmonary inflammation may also influence skeletal muscle indirectly through systemic inflammatory and metabolic responses. Time-course studies of chronic CS exposure have demonstrated that systemic inflammatory changes precede or accompany progressive alterations in skeletal muscle mass, fiber composition, and metabolic pathways, that supports an interaction between systemic inflammation and muscle remodeling during disease progression. However, because systemic inflammation, oxidative stress, direct smoke toxicity, metabolic alterations, reduced physical activity, and nutritional changes develop concurrently in CS models, the relative contribution of each mechanism to skeletal muscle dysfunction remains difficult to distinguish experimentally.

Establishing a direct lung-to-muscle signaling pathway requires evidence that pulmonary-derived mediators reach skeletal muscle and activate specific downstream molecular pathways responsible for muscle remodeling. Consequently, future studies should combine pulmonary tissue analyses with longitudinal measurements of circulating mediators and direct assessment of signaling pathway activation within skeletal muscle rather than relying solely on parallel changes in lung and muscle phenotypes.

### 2.2 Redox stress, smoking toxicity, hypoxemia, and physical inactivity

Oxidative stress represents one of the principal mechanisms linking chronic CS exposure to skeletal muscle dysfunction. Experimental studies consistently demonstrate that cigarette smoke induces oxidative modifications in both respiratory and locomotor muscles, including increased protein carbonylation, lipid peroxidation, altered nitric oxide signaling, and disruption of redox homeostasis (7, 68–70). Notably, oxidative injury develops in skeletal muscle even in the absence of substantial local inflammatory-cell infiltration, indicating that cigarette smoke may impair muscle through direct systemic or circulating redox mechanisms rather than exclusively through local inflammation (68).

Long-term CS exposure also promotes inflammatory signaling within skeletal muscle. In mouse models, chronic smoke exposure suppresses HDAC2 expression while enhancing NF-κB activation, leading to increased TNF-α and keratinocyte chemoattractant expression together with impaired myogenic differentiation. In a preclinical mouse model, chronic Cigarette smoke significantly down-regulated myosin heavy chain (MHC)-IIβ and HDAC2 expression, but enhanced NF-κBp65, keratinocyte chemoattractant (KC) and tumor necrosis factor (TNF)-α expression in the gastrocnemius muscles, suggesting that cigarette smoke inhibited HDAC2 expression and its interaction with NF-κBp65 to stimulate inflammation, contributing to the pathogenesis of COPD-related skeletal muscle atrophy in mice. Pharmacological inhibition of NF-κB largely reverses these responses, supporting a direct role for HDAC2/NF-κB signaling in cigarette smoke-induced skeletal muscle inflammation and atrophy.

Additional systemic factors further contribute to muscle dysfunction during chronic CS exposure. Experimental animals frequently exhibit reduced spontaneous physical activity, decreased food intake, body weight loss, intermittent hypoxemia, and impaired exercise tolerance, each of which independently influences skeletal muscle metabolism and protein turnover. Because these factors coexist in most CS models, skeletal muscle dysfunction should be viewed as the consequence of multiple interacting pathological processes rather than a single inflammatory pathway. Experimental studies should therefore account for differences in physical activity, nutritional status, and oxygenation when investigating mechanisms of muscle dysfunction.

### 2.3 Locomotor and respiratory muscles represent distinct phenotypes

Experimental evidence indicates that locomotor and respiratory muscles respond differently to chronic cigarette smoke exposure. Peripheral locomotor muscles, including the quadriceps, gastrocnemius, soleus, and rectus femoris, consistently exhibit reduced muscle fiber cross-sectional area, impaired contractile function, mitochondrial dysfunction, and a transition from oxidative toward glycolytic fiber types following chronic CS exposure. However, these responses vary among individual muscles, with highly oxidative muscles showing greater susceptibility to fiber-type remodeling than muscles with predominantly glycolytic characteristics.

The diaphragm exhibits a distinct pattern of remodeling. Because it remains continuously active throughout disease progression, chronic inspiratory loading induces adaptive responses that differ from those observed in peripheral skeletal muscles. Nevertheless, prolonged CS exposure produces diaphragm atrophy, oxidative stress, extracellular matrix remodeling, and altered antioxidant signaling (71). Preventive aerobic exercise (50% of maximal exercise capacity for 60 min/day, 5 days per week for 24 weeks) attenuates many of these changes in smoke exposed animals by upregulating NRF2-dependent antioxidant pathways and reducing MMP-2 and MMP-9 activity, thereby preserving diaphragm structure and function during chronic CS exposure (71).

These observations demonstrate that respiratory and locomotor muscles represent biologically distinct phenotypes and should not be interpreted interchangeably. Future mechanistic studies should therefore evaluate each muscle group independently and relate molecular alterations to corresponding structural and functional outcomes, including muscle mass, fiber cross-sectional area, contractile performance, mitochondrial function, and fatigue resistance.

## 3 Exercise regulation of skeletal-muscle proteostasis and mitochondrial function

### 3.1 Anabolic and catabolic signaling

Chronic cigarette smoke (CS) exposure disrupts skeletal muscle protein homeostasis through coordinated suppression of anabolic signaling and activation of proteolytic pathways. Reduced IGF-1/Akt/mTOR signaling, increased FoxO activity, induction of the ubiquitin-proteasome E3 ligases MuRF-1 and Atrogin-1, altered autophagy, and myostatin signaling have each been implicated, although their contribution varies by disease severity, muscle group, nutrition, and activity (64, 72–95). In BALB/c mice exposed to CS for 8 or 24 weeks, Caron et al. (65) reported reductions in gastrocnemius and soleus mass together with alterations in signaling pathways regulating protein synthesis and degradation; importantly, some of these changes persisted after smoking cessation, indicating that prolonged smoke exposure can produce sustained disturbances in muscle proteostasis (2). In another CS model, reduced nuclear translocation of serum response factor in quadriceps muscle was accompanied by downregulation of its upstream regulators STARS and RhoA and reduced Igf1 expression, providing an additional mechanism through which chronic smoke exposure may impair muscle growth and maintenance (96).

Exercise training can reverse several of these abnormalities when introduced during prolonged CS exposure. Krüger et al. exposed C57BL/6J mice to cigarette smoke for 8 months and introduced exercise training during the final 2 months (37). Smoke exposure reduced maximal oxygen uptake and running speed and induced skeletal muscle wasting, whereas exercise restored both functional outcomes. At the molecular level, training reduced activation of the ubiquitin-proteasome system, increased skeletal muscle IGF-1 expression, and reversed smoke-induced inhibition of mTOR signaling, demonstrating coordinated effects on both catabolic and anabolic pathways (37).

Additional mechanistic studies indicate that CS alters myokine-related pathways involved in muscle growth. In a mouse model characterized by emphysematous lung injury and skeletal muscle dysfunction, chronic CS exposure reduced PGC-1α and FNDC5 expression while increasing myostatin, phosphorylated ERK1/2, and phosphorylated SMAD3 in skeletal muscle (15). Complementary C2C12 experiments showed that inhibition of ERK1/2 or SMAD3 partially restored FNDC5 expression, supporting a myostatin–ERK/SMAD3–PGC-1α pathway linking smoke exposure to impaired muscle homeostasis (15).

Collectively, these studies indicate that smoke-induced muscle wasting is accompanied by simultaneous inhibition of anabolic signaling and activation of several catabolic pathways rather than by alteration of a single molecular target. The therapeutic exercise model of Krüger et al. (8, 37) is particularly informative because exercise was introduced after prolonged smoke exposure and restored both molecular markers of protein homeostasis and whole-body exercise capacity.

### 3.2 Oxidative stress and mitochondrial remodeling

Mitochondrial dysfunction is a reproducible feature of locomotor muscle in COPD, with reduced oxidative enzyme activity, altered mitochondrial density/function, and greater ROS burden reported in clinical and experimental studies (2, 3, 97–103). CS exposure produces marked alterations in skeletal muscle oxidative metabolism. In mice exposed to cigarette smoke for 8 or 16 weeks, PGC-1α expression decreased by approximately 48% in soleus and 23% in extensor digitorum longus muscle (104). These changes were accompanied by reductions in citrate synthase activity of approximately 19% and 17%, respectively, reductions in β-hydroxyacyl-CoA dehydrogenase activity, capillary regression in soleus muscle, and impaired exercise endurance. Serum TNF-α increased substantially during smoke exposure, and TNF-α treatment of myoblasts suppressed PGC-1α expression, providing a potential mechanistic link between systemic inflammatory signaling and impaired oxidative phenotype (104).

More direct assessments of mitochondrial bioenergetics confirm these abnormalities. In emphysematous mice chronically exposed to CS, ATP synthesis in gastrocnemius muscle was reduced together with muscle-fiber cross-sectional area and the COX4-to-citrate synthase activity ratio, indicating an early bioenergetic defect in locomotor muscle (105). Similarly, 8 months of CS exposure increased skeletal muscle 4-hydroxynonenal approximately twofold and reduced respiratory-chain and uncoupling-protein abundance, while also altering substrate-supported mitochondrial respiration (106). Acute exposure experiments further demonstrate that cigarette smoke constituents can directly impair mitochondrial electron transport, particularly complex I-supported respiration, in both fast- and slow-twitch skeletal muscle fibers (107).

Exercise attenuates several of these smoke-induced metabolic abnormalities. Exercise also activates PGC-1alpha-dependent programs involved in mitochondrial biogenesis and oxidative metabolism, increases antioxidant defenses, and can reduce reliance on glycolysis at a given workload. These adaptations provide a plausible mechanism for improved endurance and reduced activation of redox-sensitive proteolytic pathways. The response is not uniform. COPD muscle may show incomplete mitochondrial adaptation despite training, and different exercise modes elicit different signals: endurance training strongly targets oxidative capacity, whereas resistance training provides a more direct hypertrophic/mechanical stimulus. The statement that ‘exercise improves mitochondrial function’ should therefore be qualified by exercise modality, tissue, and endpoint.

In the therapeutic model of Krüger et al., exercise introduced during the final 2 months of an 8-month CS exposure improved skeletal muscle oxidative capacity and metabolism in parallel with restoration of maximal oxygen uptake and running performance (37). In a separate time-course study, Toledo-Arruda et al. exposed mice to CS and aerobic exercise for 4, 8, or 12 weeks and found that training improved functional capacity and attenuated the CS-induced increase in cachexia index after 12 weeks. The study further demonstrated exercise-dependent modulation of skeletal muscle oxidative and antioxidant pathways, including Nrf2-related signaling (31).

Respiratory muscle studies show a similar interaction between exercise and redox regulation. In mice exposed to cigarette smoke for 24 weeks, aerobic training prevented diaphragm wasting, increased expression of the antioxidant genes Nrf2 and Hmox1, reduced MMP-2 and MMP-9 activity, and improved aerobic capacity relative to smoke-exposed sedentary animals (71). These findings indicate that the mitochondrial and redox response to exercise is muscle-specific and involves coordinated regulation of bioenergetics, antioxidant defense, and extracellular matrix remodeling.

### 3.3 Integration of molecular and functional adaptations

The available animal studies demonstrate that molecular adaptations to exercise are accompanied by measurable improvements in muscle and whole-body phenotype. In the Krüger model, exercise not only restored IGF-1/mTOR signaling and reduced ubiquitin-proteasome activation but also improved skeletal muscle structure, oxidative capacity, maximal oxygen uptake, and maximal running speed (37). This parallel improvement in molecular and functional outcomes provides stronger evidence of physiological recovery than changes in signaling proteins alone.

Similarly, Toledo-Arruda and colleagues demonstrated that aerobic exercise during CS exposure improved functional capacity while attenuating skeletal muscle oxidative stress and cachexia-related changes over time (31). In the diaphragm, exercise-induced upregulation of antioxidant signaling and suppression of MMP activity were accompanied by preservation of muscle mass and aerobic performance (71). Together, these findings indicate that exercise-induced modulation of proteostasis, mitochondrial function, and redox balance translates into measurable preservation or recovery of muscle phenotype in CS-exposed animals.

Nevertheless, the magnitude and nature of these adaptations depend on the muscle examined and the timing of exercise. Preventive paradigms, in which training occurs concurrently with CS exposure, primarily demonstrate attenuation of disease development, whereas the Krüger model provides evidence that exercise initiated after prolonged smoke exposure can partially restore established skeletal muscle abnormalities. This distinction is essential when interpreting exercise as a modifier of COPD-associated skeletal muscle dysfunction.

## 4 Muscle-to-lung signaling: candidate mediators and strength of evidence

### 4.1 Myokines and cytokines

Skeletal muscle releases a broad number of secreted mediators during contraction, but not every exercise-responsive mediator in blood is muscle-derived. IL-6 is the clearest example of context dependence. Contracting muscle can release IL-6 acutely as a metabolic signal that contributes to glucose mobilization and can precede increases in anti-inflammatory mediators such as IL-1ra and IL-10. Chronic elevation of IL-6 in inflammatory disease, however, is associated with adverse systemic phenotypes and disease outcomes (108–116). Thus, IL-6 should not be labeled simply as pro- or anti-inflammatory without specifying source, concentration, timing, and physiological context.

Irisin (cleaved from FNDC5) is a candidate muscle-derived signal with more direct COPD-model evidence. In a 12-week CS model, treadmill exercise was performed before daily smoke exposure and was associated with greater irisin and Nrf2 signaling and attenuation of emphysematous changes (47). This supports an exercise-irisin-Nrf2 association in a concurrent/preventive design. It does not by itself prove that skeletal-muscle-derived irisin is necessary or sufficient for the pulmonary effect; tissue-specific FNDC5 manipulation or irisin-receptor blockade would provide stronger causal evidence.

IL-10 and IL-1ra are important anti-inflammatory mediators but are predominantly produced by immune cells and other tissues rather than functioning as canonical muscle-specific myokines. Exercise can increase these mediators systemically, and human COPD training studies suggest changes in regulatory immune balance (44, 110–116). Their role in muscle-lung communication is therefore best described as part of an exercise-modified systemic immune environment unless muscle origin is directly demonstrated.

Human COPD training studies further illustrate why source and exercise modality must be specified. Resistance-training interventions have measured circulating IL-6, TNF-alpha, IL-10, and IL-15 together with strength and metabolic outcomes, but changes in plasma cytokines cannot establish skeletal muscle as their source (117). Similarly, direct comparisons of endurance and resistance training demonstrate that both modalities can improve function while producing partly different local muscle remodeling and inflammatory/redox responses (118, 119). Thus, circulating cytokine changes should be interpreted as systemic exercise responses unless paired with muscle expression, arteriovenous balance, tissue-specific tracing, or another source-resolving approach.

IL-15 is a useful example of a candidate myokine whose biological plausibility exceeds the current evidence for lung targeting. IL-15 is expressed in skeletal muscle and has been linked to muscle phenotype, oxidative metabolism, and protein turnover; in an experimental COPD model, manipulation of IL-15 was associated with changes in ubiquitin-proteasome-related muscle protein degradation (23). However, direct evidence that exercise-induced muscle-derived IL-15 reaches the lung and modifies pulmonary inflammation in COPD is lacking. IL-15 should therefore be considered a candidate muscle-regulatory signal rather than an established mediator of muscle-to-lung crosstalk.

### 4.2 Metabolite signaling

Exercise changes circulating metabolites that can alter immune-cell function, creating another potential route of muscle-lung communication. Lactate can signal through hydroxycarboxylic acid receptor 1 (HCAR1/GPR81) and modify inflammatory-cell metabolism; depending on concentration, cell type, and microenvironment, lactate can suppress or redirect inflammatory responses (120–124). However, direct evidence that muscle-derived lactate mediates a lung benefit in CS-COPD is currently lacking. Its role should therefore be considered plausible but unproven.

NAD+ metabolism and TCA-cycle intermediates such as succinate, citrate, and itaconate regulate sirtuins, HIF-1alpha, Nrf2, and immunometabolic programs (125–131). These pathways are relevant to both muscle and lung biology, but most mechanistic evidence comes from cellular immunometabolism or non-COPD contexts. A key unresolved question is whether exercise changes the circulating concentration or tissue flux of these metabolites enough to alter pulmonary immune-cell behavior in COPD.

Lactate illustrates the difference between pathway plausibility and demonstrated inter-organ causality. Exercise can produce large, transient increases in circulating lactate, and lactate can signal through HCAR1/GPR81 and through lactate-dependent changes in cellular metabolism and protein lactylation. In lung models outside COPD, locally produced lactate can suppress inflammatory alveolar-macrophage activation, demonstrating that pulmonary immune cells are capable of responding to lactate-related signals (132). However, this does not establish that exercise-derived skeletal-muscle lactate is the relevant source in COPD. A definitive test would require simultaneous muscle and plasma lactate flux measurements, lung-cell HCAR1 pathway engagement, and receptor blockade or isotope tracing during exercise in a CS model.

TCA-cycle metabolites also warrant source-specific interpretation. Succinate can amplify inflammatory signaling through HIF-1alpha and related pathways, whereas itaconate can restrain macrophage inflammatory programs and activate Nrf2-dependent antioxidant responses. Recent CS-COPD work showing that the itaconate derivative 4-octyl itaconate attenuated airway inflammation and emphysematous injury through Nrf2-dependent effects in alveolar macrophages strengthens the relevance of this pathway to COPD lung biology (133). Nevertheless, pharmacological delivery of an itaconate derivative is not evidence that exercising muscle supplies endogenous itaconate to the lung. The key crosstalk question is therefore not whether these metabolites can regulate pulmonary immunity, but whether exercise alters their inter-organ flux sufficiently to do so *in vivo*.

NAD+ biology should be interpreted similarly. Exercise-associated improvements in muscle mitochondrial redox state and NAD+-dependent sirtuin signaling may reduce oxidative stress and alter the circulating metabolome, yet evidence for a discrete muscle-derived NAD+ signal acting directly on the COPD lung is currently insufficient. For this mediator class, targeted metabolomics alone is unlikely to establish mechanism; stable-isotope flux analysis, tissue-specific enzyme manipulation, and demonstration of target-cell pathway engagement would provide substantially stronger evidence.

### 4.3 Extracellular vesicles: lung-derived and exercise-associated signals

EVs provide a physically plausible vehicle for bidirectional signaling because they carry proteins, lipids, and nucleic acids and can be taken up by distant cells. In the lung-to-systemic direction, airway epithelial cells exposed to cigarette-smoke extract can release EVs that alter macrophage inflammatory programs; for example, epithelial-cell EV cargo has been linked to pro-inflammatory macrophage responses in experimental systems (134–136). These observations support lung-derived EV signaling, although their contribution to skeletal-muscle dysfunction *in vivo* remains poorly defined.

In the muscle-to-lung direction, acute and chronic exercise alter circulating EV abundance and cargo, but circulating EVs originate from multiple cell types, including platelets, leukocytes, endothelium, and skeletal muscle (137–149). Consequently, an increase in plasma EVs after exercise cannot be assigned to muscle without source tracing. Evidence that exercise-associated EVs directly reach the lung and modify COPD pathology is currently limited, and claims of anti-inflammatory pulmonary EV action should be framed as a hypothesis until transfer, depletion, or cell-specific tracing studies establish causality.

Candidate EV cargo relevant to crosstalk includes miRNAs, mitochondrial/metabolic proteins, redox-regulatory proteins, and inflammatory mediators. The most informative future studies would combine tissue-specific EV labeling with proteomic/RNA cargo profiling, lung uptake mapping, and functional transfer into smoke-exposed recipients. Statements based on unpublished or abstract-only datasets have been removed from the mechanistic argument of this review.

Specific cargo studies strengthen the evidence that EVs can mediate local cell-to-cell signaling in smoke-exposed lungs. CSE-stimulated bronchial epithelial cells have been reported to release exosomal miR-221-3p that promotes M1 macrophage polarization through SOCS3/STAT3 signaling (150). Other COPD-focused work has implicated epithelial exosomal miR-125a-5p in macrophage polarization through IL1RN (151). These findings provide mechanistic support for lung-derived EV signaling, but they primarily describe epithelial-to-macrophage communication within the pulmonary compartment rather than lung-to-muscle communication.

Evidence for muscle-derived EV signaling is stronger in non-pulmonary target organs than in COPD lung. In a mechanical-overload model, skeletal muscle released EVs enriched in miR-1, and inhibition of EV release prevented downstream adipose-tissue signaling, providing a source-to-cargo-to-target proof of principle for muscle-derived EV communication (152). Conversely, human exercise studies show that circulating EV miRNA profiles do not necessarily mirror skeletal-muscle miRNA expression, emphasizing that plasma EV cargo cannot be assigned to muscle without source validation (153). Therefore, candidate muscle-to-lung EV mechanisms in COPD should require tissue-of-origin labeling, demonstration of pulmonary uptake, and gain/loss-of-function experiments before a specific EV cargo is considered causal.

### 4.4 Mitochondrial damage-associated molecular patterns

Mitochondrial damage-associated molecular patterns (mtDAMPs), including mitochondrial DNA (mtDNA), N-formyl peptides, and cardiolipin, are normally sequestered within mitochondria but can initiate innate immune responses when released into the cytosol or extracellular environment following mitochondrial stress or cellular injury (154–161). mtDNA can activate several pattern-recognition pathways, including TLR9 and the cGAS–STING pathway, while oxidized mtDNA can promote NLRP3 inflammasome activation and subsequent maturation of IL-1β and IL-18. N-formyl peptides released from damaged mitochondria can activate formyl peptide receptors, particularly FPR1, promoting neutrophil chemotaxis and oxidative burst, whereas redistribution of cardiolipin from the inner to the outer mitochondrial membrane can facilitate NLRP3 inflammasome assembly and activation (162).

Evidence from cigarette smoke (CS) models directly implicates mitochondrial injury and mtDNA release in COPD-related inflammation (163). In mice exposed to CS for 6 months, emphysematous remodeling was accompanied by an almost three-fold increase in circulating cell-free mtDNA compared with room-air controls (163). Complementary experiments in human bronchial epithelial cells demonstrated that CSE reduced mitochondrial membrane potential, increased oxidative stress, altered mitochondrial dynamics, and promoted extracellular mtDNA release, including the release of mtDNA within EVs (163). These responses occurred in parallel with increased expression of the DNA sensors cGAS and NLRP3 and inflammatory mediators including IL-1β, IL-6, IL-18, and CXCL2, providing a mechanistic link between CS-induced mitochondrial damage, mtDNA release, and inflammatory signaling (163). Experimental studies further indicate that CS-induced mitochondrial dysfunction can activate NLRP3-dependent inflammatory signaling in the lung, while disruption of NLRP3 attenuates CS-induced airway inflammation. CS-associated DNA sensing through cGAS–STING has also been implicated in type I interferon-dependent pulmonary inflammation, although the contribution of STING appears to vary according to experimental context (163).

These findings establish that mtDAMPs, particularly mtDNA, could be recognized as potential mediators linking mitochondrial injury to innate immune activation in CS-induced COPD. Their contribution to muscle-lung crosstalk, however, remains less clearly defined. Exercise training promotes skeletal muscle mitochondrial quality control through coordinated regulation of mitochondrial biogenesis, mitophagy, antioxidant defenses, and mitochondrial dynamics (159, 164). In CS-related experimental models, preservation of parkin-mediated mitophagy limits the accumulation of damaged mitochondria and mitochondrial ROS in skeletal muscle, while muscle contraction can preserve Parkin expression under CSE exposure (165). These adaptations could theoretically reduce the release of mtDAMPs from damaged skeletal muscles mitochondria. However, direct evidence that exercise training could reduce muscle-derived mtDAMP release in CS-induced COPD has not yet been stablished.

## 5 Exercise as a modifier of bidirectional muscle-lung crosstalk

### 5.1 Pulmonary inflammatory and redox responses in experimental models

Aerobic exercise training attenuates several components of CS-induced pulmonary inflammation and oxidative injury in animal models, although the magnitude of protection depends on the duration and timing of training and CS exposure. In an early study using C57BL/6 mice exposed to CS for 24 weeks, Toledo et al. (30) demonstrated that concurrent moderate-intensity aerobic training attenuated the CS-induced increase in mean linear intercept and decline in pulmonary elastance (30). Exercise training also reduced reactive oxygen species (ROS) in BAL fluid and 8-isoprostane expression in lung tissue, while preventing CS-induced reductions in IL-10, tissue inhibitor of metalloproteinase-1 (TIMP-1), and CuZn-superoxide dismutase and increasing glutathione peroxidase-positive cells (30). These findings indicate that exercise simultaneously modified inflammatory, oxidative, and tissue-remodeling responses during the development of CS-induced emphysema.

Subsequent time-course experiments further characterized the development of this protective response. Toledo-Arruda et al. (36), this time, exposed mice to CS with or without aerobic training for 4, 8, or 12 weeks and demonstrated progressive recruitment of inflammatory cells into BAL fluid, with macrophage accumulation evident after 4 weeks, increased neutrophils and lymphocytes after 8 weeks, and increased lymphocytes and macrophages after 12 weeks (36). Exercise training attenuated inflammatory-cell accumulation and, after 12 weeks, protected against emphysematous enlargement and reductions in tissue damping and elastance (36). These pulmonary effects were accompanied by increased IL-6 and IL-10 in both quadriceps muscle and lung, increased pulmonary total antioxidant capacity and superoxide dismutase activity, and reduced pulmonary TNF-α and oxidant levels (36). Notably, protection became more evident after adaptation to the exercise protocol, suggesting that the pulmonary response develops progressively rather than representing an immediate consequence of exercise exposure.

Other experimental studies support suppression of inflammatory signaling as an additional mechanism. In mice trained before and during subsequent environmental CS exposure, exercise training attenuated CS-induced bronchoalveolar-capillary permeability, inflammatory-cell infiltration, epithelial thickening, and expression of inflammatory cytokines, chemokines, and adhesion molecules (45). These changes were accompanied by reduced activation of pulmonary NF-κB, linking prior exercise conditioning with suppression of a major inflammatory signaling pathway during subsequent smoke exposure. Given that exercise training was initiated before CS exposure, these findings reflect protection against the development of CS-induced pulmonary pathology rather than reversal of established diseases.

Exercise-mediated pulmonary protection may also involve neuroimmune regulation. In a 12-week CS model, aerobic treadmill training prevented emphysematous enlargement and reduced mononuclear-cell infiltration in wild-type mice (166). Suehiro and colleagues examined the contribution of cholinergic signaling using mice with a 65-70% reduction in vesicular acetylcholine transporter (VAChT) expression. The protective effects of exercise on emphysema and mononuclear-cell infiltration observed in wild-type animals were absent in VAChT-deficient mice, whereas exercise reduced iNOS-positive cells and collagen deposition in both genotypes (166). These findings provide mechanistic evidence that intact cholinergic signaling contributes, at least in part, to specific anti-inflammatory and structural effects of aerobic exercise in CS-exposed lungs rather than serving merely as an accompanying systemic response.

Importantly, pulmonary structural responses to exercise are not uniform across experimental models. In mice exposed to CS and subsequently subjected to four weeks of interval training, exercise increased peak oxygen uptake but did not reduce the CS-induced increase in alveolar airspace size (167). Similarly, in a 20-week smoke-exposure model, six weeks of high-intensity interval treadmill training improved exercise capacity and attenuated smoke-induced cardiac, endothelial, and diaphragm dysfunction, despite the absence of overt airway destruction in the experimental model (168). Exercise reduced the smoke-induced increases in diaphragm oxidative stress and protein degradation, demonstrating that substantial extrapulmonary adaptation can occur independently of measurable reversal of structural lung disease.

Therapeutic exercise approaches provide further evidence that the effects of training extend beyond prevention. Krüger et al. (37) exposed mice to CS for 8 months and introduced exercise only during the final two months. Exercise training restored maximal oxygen uptake and running speed, reduced circulating inflammatory cytokines and T-cell adhesion molecule expression, and improved skeletal muscle structure, oxidative capacity, and anabolic signaling (37). Although this study primarily characterized systemic and skeletal muscle rather than pulmonary structural outcomes, it demonstrates that exercise initiated after prolonged CS exposure can reverse important extrapulmonary consequences of chronic smoke exposure.

Collectively, these studies demonstrate that aerobic exercise can attenuate CS-induced pulmonary inflammation and oxidative stress through several pathways, including reduced inflammatory-cell recruitment, suppression of NF-κB-associated signaling, enhancement of antioxidant defenses, modulation of inflammatory cytokines, and cholinergic regulation. Under concurrent or preventive training conditions, these responses can be accompanied by preservation of lung mechanics and attenuation of emphysematous remodeling. However, the available evidence does not support a uniform reversal of structural lung disease by exercise, particularly when training is initiated after prolonged CS exposure. The distinction between prevention of CS-induced pulmonary injury and treatment of established disease is therefore critical when considering exercise as a modifier of muscle-lung crosstalk.

### 5.2 Exercise modality and training stimulus matter

The biological response to exercise training is determined by the characteristics of the training stimulus, including modality, intensity, duration, frequency, and timing relative to CS exposure. This distinction is particularly important when evaluating muscle- lung crosstalk because different exercise programs impose different metabolic and mechanical demands on skeletal muscle and may therefore generate distinct profiles of myokines, metabolites, extracellular vesicles, and other circulating signals. However, direct comparisons of exercise modalities within the same experimental COPD model remain limited.

Most preclinical studies of CS-induced COPD have used moderate-intensity treadmill training (31, 34, 36). In mice exposed concurrently to CS and treadmill exercise for 4, 8, or 12 weeks, the pulmonary response developed progressively with training duration (36). While exercise did not substantially attenuate CS-induced pulmonary alterations after 4 weeks, the accumulation of inflammatory cells was reduced after 8 weeks in BAL fluid (36). After 12 weeks, exercise increased pulmonary IL-10, glutathione, and superoxide dismutase activity, reduced TNF-α and oxidative stress, attenuated pulmonary inflammation, and improved lung mechanics (36). These findings suggest that the duration of training and development of exercise adaptation influence the pulmonary response to exercise in CS-exposed animals.

Training intensity may also influence the response. High-intensity interval training (HIIT) has been examined in chronic CS-exposed mice using protocols in which training was introduced during ongoing smoke exposure. In mice exposed to CS for 14 weeks, four weeks of HIIT improved exercise capacity and attenuated smoke-induced right ventricular dysfunction and pulmonary vascular remodeling, although it did not reduce CS-induced alveolar enlargement (167). In a separate model involving 20 weeks of CS exposure, six weeks of HIIT improved exercise capacity and several extrapulmonary outcomes, including diaphragm function and mitochondrial adaptations, despite the absence of overt smoke-induced airway destruction in that model (168). Transcriptomic analysis of the same experimental approach subsequently identified exercise-CS interactions in pulmonary and cardiovascular gene expression. This further indicates that interval training can modify tissue-specific responses to chronic smoke exposure.

Although very limited studies available, resistance-type exercise produces a different skeletal-muscle stimulus in animal model of COPD. In Wistar rats exposed to secondary cigarette smoke for 16 weeks, progressive-load resistance training consisting of repeated ladder climbing prevented smoke-induced alterations in gastrocnemius mitochondrial function (169). Smoke exposure reduced respiratory-chain proteins representing complexes I, II, IV, and V and altered mitochondrial oxygen consumption, whereas these abnormal responses were attenuated in resistance-trained smoke-exposed animals (169). This study demonstrates that resistance exercise can preserve skeletal-muscle mitochondrial homeostasis during chronic smoke exposure; however, pulmonary outcomes and circulating mediators were not assessed, which prevents conclusions regarding muscle-to-lung signaling.

Exercise timing represents an additional source of heterogeneity. Some experimental studies initiate training before CS exposure and therefore primarily examine protection against subsequent smoke-induced injury. For example, prior exercise conditioning attenuated CS-induced pulmonary permeability, inflammatory-cell infiltration, epithelial remodeling, and NF-κB activation during subsequent smoke exposure (45). Other studies initiate exercise after pulmonary pathology has begun to develop. In a 90-day CS model, treadmill training introduced after 60 days of smoke exposure reduced BAL and circulating inflammatory-cell accumulation, decreased IL-1β, CXCL1, IL-17, and TNF-α, increased IL-10, and modulated pulmonary STAT3 signaling (34). These differences in experimental design are important because prevention of disease development and modification of established pathology represent biologically distinct effects of exercise.

Collectively, the available preclinical evidence indicates that exercise should not be considered a uniform biological stimulus in experimental COPD model. Training duration, intensity, modality, and timing relative to CS exposure influence pulmonary, systemic, and skeletal-muscle adaptations. Importantly, direct head-to-head comparisons of endurance, interval, and resistance training within the same CS-induced COPD model are currently lacking. Such studies, incorporating simultaneous measurements of skeletal-muscle adaptation, circulating mediators, and pulmonary outcomes, would help determine whether specific exercise stimuli generate distinct muscle-derived signals capable of modifying lung pathology.

### 5.3 From association to causality

A rigorous crosstalk experiment requires more than parallel improvement in muscle and lung. At minimum, the candidate mediator should change with CS and/or exercise, its source should be identified, the target lung cell or receptor should be defined, and manipulation of the mediator should alter a pulmonary phenotype. Strong evidence would include muscle-specific knockout/overexpression, neutralization or receptor blockade, parabiosis or adoptive transfer, tissue-specific EV tracing/depletion, or rescue experiments. Human translation would then ask whether the same mediator changes with pulmonary rehabilitation and associates with prespecified muscle and lung outcomes.

An evidence hierarchy can therefore be applied. The lowest level is temporal association: exercise changes a circulating factor while lung and muscle outcomes improve. A stronger level demonstrates source and target engagement, for example increased production in contracting muscle together with receptor activation in a defined lung-cell population. Necessity is established when blocking the mediator, its release, or its receptor diminishes the pulmonary effect of exercise; sufficiency is established when transfer or replacement reproduces part of the phenotype in the absence of training. Rescue experiments that restore the effect after genetic or pharmacological interruption provide particularly persuasive evidence. This hierarchy should be applied separately to each mediator rather than to the muscle-lung concept as a whole.

Human studies cannot usually perform equivalent genetic manipulations, but they can strengthen translation through repeated time-resolved sampling, arteriovenous measurements across exercising muscle, muscle biopsy, EV source markers, targeted metabolomics, and prespecified pulmonary outcomes. Mediation analysis may then test whether changes in a candidate signal statistically account for part of the training response, while acknowledging that statistical mediation does not itself prove biological causality. Convergence between such human data and source-specific perturbation experiments in CS models would provide the strongest basis for identifying clinically relevant muscle-lung pathways.

## 6 Experimental priorities and recommended measurements

Future studies should align experimental measurements with a defined causal sequence: source tissue → circulating mediator → target lung cell/pathway → pulmonary phenotype. Demonstrating parallel improvements in skeletal muscle and lung after exercise is insufficient to establish inter-organ communication. Instead, candidate mediators should be traced from their tissue of origin to the circulation and ultimately to a defined biological response in the target organ. This approach would distinguish direct muscle-lung signaling from independent adaptations of both tissues to exercise.

### 6.1 Source and muscle phenotype

Locomotor and respiratory muscles should be analyzed separately because their responses to CS exposure and exercise differ substantially. Muscle-derived signaling should first be supported by evidence of adaptation within the proposed source tissue. Relevant endpoints include muscle mass and fiber cross-sectional area (CSA), force and fatigue resistance, fiber-type distribution and capillary density, and direct assessment of protein synthesis when feasible using puromycin/SUnSET or stable-isotope approaches. Molecular analyses should include Akt/mTOR and FoxO–MuRF-1/Atrogin-1 signaling, mitochondrial respiration and oxidative capacity, PGC-1α/TFAM signaling, oxidative damage, and markers of autophagy and mitophagy. In CS models, spontaneous activity, food intake, body weight, and oxygenation should also be measured because changes in these variables can independently influence skeletal muscle phenotype and confound interpretation of lung-to-muscle signaling.

### 6.2 Circulating mediator and source attribution

Measurement of a circulating mediator alone does not establish its tissue of origin. Candidate biomarker should therefore be assessed simultaneously in skeletal muscle and plasma, with muscle-specific genetic manipulation or arteriovenous approaches adapted to experimental models where feasible. EV studies should report particle concentration and size together with established EV markers and cargo characterization. Tissue-specific labeling, genetic reporter systems, or validated surface markers would provide stronger evidence that circulating EVs originate from skeletal muscle and subsequently reach pulmonary target cells. Similarly, measurements of metabolites and mtDAMPs should incorporate time-resolved sampling and corresponding muscle mitochondrial/metabolic phenotyping. These approaches are particularly important for mediators such as IL-6, EVs, and mtDNA that can originate from multiple tissues during CS exposure and exercise.

### 6.3 Lung target and phenotype

Evidence for muscle-to-lung signaling requires demonstration that a circulating mediator reaches or influences a defined pulmonary target. Candidate target cells include airway and alveolar epithelial cells, macrophages, neutrophils, endothelial cells, and fibroblasts, depending on the mediator investigated. Target engagement can be evaluated through receptor activation and downstream pathways such as Nrf2, NF-κB, STAT3, NLRP3, or cGAS–STING. These molecular endpoints should be accompanied by pulmonary phenotypes, including BAL inflammatory-cell counts and cytokines, oxidative-stress markers, epithelial or macrophage mitochondrial function, histological emphysema indices such as mean linear intercept and destructive index, airway remodeling, lung mechanics, and host-defense outcomes in exacerbation models. Changes in signaling pathways without corresponding structural, inflammatory, or functional outcomes should be interpreted as pathway modulation rather than evidence of disease modification.

### 6.4 Experimental tests of necessity and sufficiency

Establishing causality represents the major experimental priority for the muscle-lung crosstalk hypothesis. Necessity could be tested using muscle-specific deletion or silencing of a candidate myokine, inhibition of its release, or pharmacological/genetic blockade of its receptor in pulmonary target cells. If the pulmonary benefits of exercise are diminished despite preservation of the exercise stimulus, this would provide evidence that the mediator contributes to the response. Sufficiency could be examined by administering the candidate mediator or transferring muscle-derived EVs from exercised animals to sedentary CS-exposed recipients and determining whether pulmonary effects are reproduced in the absence of training.

EV and metabolite studies require particularly rigorous source tracing. Purified EVs from trained and sedentary animals should be compared using matched particle numbers and validated tissue-of-origin markers, while tissue-specific inhibition of EV biogenesis or release could determine whether skeletal muscle-derived vesicles are necessary for pulmonary adaptation. Genetic labeling or isotope tracing could similarly establish whether exercise-responsive metabolites or mitochondrial products released from skeletal muscle enter pulmonary cells. Combining source tracing with receptor blockade and rescue experiments would provide the strongest evidence for a defined skeletal muscle → circulating mediator → lung target → pulmonary phenotype pathway.

## 7 Conclusions

Chronic CS exposure produces concurrent pulmonary and skeletal muscle abnormalities through interconnected inflammatory, oxidative, metabolic, and mitochondrial processes. Experimental studies further demonstrate that exercise training can attenuate several of these responses, including pulmonary and systemic inflammation, oxidative stress, skeletal muscle wasting and mitochondrial dysfunction, and, in selected models, emphysematous remodeling and impaired exercise capacity. Together, these findings provide a biological basis for bidirectional muscle-lung communication and support its investigation as a mechanistic framework for understanding the systemic effects of exercise in COPD.

However, direct evidence linking specific signals originating from skeletal muscle to pulmonary responses remains limited. Many exercise studies employ preventive or concurrent training programs, the tissue origin of circulating mediators is frequently unresolved, and evidence for several proposed mediators derives from associative or complementary experimental models. Myokines, metabolites, extracellular vesicles, and mitochondrial stress signals therefore represent candidate mediators rather than established components of a causal muscle-lung pathway.

Future studies should move beyond parallel assessment of muscle and lung responses and establish the source, target, necessity, and sufficiency of individual mediators using tissue-specific manipulation, source tracing, receptor blockade, and transfer approaches. Such studies will determine whether exercise-responsive signals from skeletal muscle directly modify pulmonary pathology and identify the pathways that contribute to the systemic benefits of exercise in experimental COPD.
